# Transforming the study of organisms: Phenomic data models and knowledge bases

**DOI:** 10.1371/journal.pcbi.1008376

**Published:** 2020-11-24

**Authors:** Anne E. Thessen, Ramona L. Walls, Lars Vogt, Jessica Singer, Robert Warren, Pier Luigi Buttigieg, James P. Balhoff, Christopher J. Mungall, Deborah L. McGuinness, Brian J. Stucky, Matthew J. Yoder, Melissa A. Haendel

**Affiliations:** 1 Environmental and Molecular Toxicology, Oregon State University, Corvallis, Oregon, United States of America; 2 Ronin Institute for Independent Scholarship, Monclair, New Jersey, United States of America; 3 Bio5 Institute, University of Arizona, Tucson, Arizona, United States of America; 4 TIB Leibniz Information Centre for Science and Technology, Hannover, Germany; 5 Annex Agriculture Inc., Saskatchewan, Canada; 6 Alfred-Wegener-Institut, Helmholtz-Zentrum für Polar- und Meeresforschung, Bremerhaven, Germany; 7 Renaissance Computing Institute, University of North Carolina, Chapel Hill, North Carolina, United States of America; 8 Environmental Genomics and Systems Biology, Lawrence Berkeley National Laboratory, Berkeley, California, United States of America; 9 Rensselaer Polytechnic Institute, Troy, New York, United States of America; 10 Florida Museum of Natural History, University of Florida, Gainesville, Florida, United States of America; 11 Illinois Natural History Survey, Champaign, Illinois, United States of America; CNRS, FRANCE

## Abstract

The rapidly decreasing cost of gene sequencing has resulted in a deluge of genomic data from across the tree of life; however, outside a few model organism databases, genomic data are limited in their scientific impact because they are not accompanied by computable phenomic data. The majority of phenomic data are contained in countless small, heterogeneous phenotypic data sets that are very difficult or impossible to integrate at scale because of variable formats, lack of digitization, and linguistic problems. One powerful solution is to represent phenotypic data using data models with precise, computable semantics, but adoption of semantic standards for representing phenotypic data has been slow, especially in biodiversity and ecology. Some phenotypic and trait data are available in a semantic language from knowledge bases, but these are often not interoperable. In this review, we will compare and contrast existing ontology and data models, focusing on nonhuman phenotypes and traits. We discuss barriers to integration of phenotypic data and make recommendations for developing an operationally useful, semantically interoperable phenotypic data ecosystem.

## Author summary

Organism traits determine the role of species in economies and ecosystems, and the expression of those traits relies on interactions between an organism’s genes and environment. The key to predicting trait expression is having a large pool of data to derive models, but most organism trait observations are recorded in ways that are not computational. In this paper, intended for an interdisciplinary audience, we discuss data models for representing organism traits in a computable format. Increasing acceptance of a data model for traits will greatly increase the pool of available data for studying the dynamic processes that determine trait expression. We hope that explaining these data models in a straightforward way and articulating their potential for accelerating discovery will increase adoption of this promising data standard.

## Introduction

An organism’s phenotype is the product of interactions between its genetic endowment and environmental conditions over its lifetime, but the ability to predict phenotypes from genotype and environmental data is limited. The models we currently have that predict organism phenotype from genotype rarely include environments, have lower performance on multigene phenotypes, and often only apply to a single taxon [1–18]. The majority of existing models that do include environmental change focus on ecosystems and are driven entirely by environmental data and organism abundance/distribution (see [19,20] for example). Genes and phenotypes are assumed present using species observations or environmental measurements of biological activity. The models are typically very geospatially specific, predicting results for a single system, such as the Chesapeake Bay [21]; moreover, they reveal more about the physical, chemical, and ecological processes happening in that system than they do about organism phenotypes. Such models can tell us what to expect in different scenarios and can be used to probe specific parts of the ecosystem. We need an analogous model for predicting and explaining phenotypic changes in organisms.

Worldwide recognition of climate change has created urgency around addressing this problem for agricultural sustainability and conservation of essential ecosystem functions [22]. Models for deriving phenotypic characteristics do not have access to sufficient gene, environment, and phenotypic data to make accurate predictions at the organism or population levels, especially outside humans and model organisms. The problem is not only merely a lack of data but also that extant data cannot be combined at scale, especially for phenotype and environment data that have a strong temporal component [23,24]. A mechanism to scale up data integration is needed if we aim to have a data set large enough to predictively model the relationship between phenotypes, genotypes, and environments. In this review, we describe the barriers to large-scale integration of phenotypic data, compare and contrast existing semantic data models, and provide best practices for representing characteristics of organisms using data models with explicit semantics. Although some of the methods we describe have their origin in biomedical research, others have arisen in the ecology, evolution, and biodiversity communities as a result of the particular data challenges that come with describing phenotypes of tens or thousands of species. Work on integrating phenotypic data for humans and model organisms is reviewed elsewhere [25–30].

Information about organismal phenotypes has been collected by thousands of observers for a myriad of purposes over centuries. Much of this information is contained in countless small, heterogeneous data sets [31], which are not findable, accessible, interoperable, reusable, traceable, licensed, or connected [32]. As a consequence, so much manual work is needed to integrate and normalize these data that it is very rarely done. One way to attack this problem is to employ a standard for describing and exchanging information about phenotypes [23,33]. Several discipline- or taxon-specific databases have been developed in an effort to make these many smaller phenotypic data sets available and reusable (e.g., [34,35]), but even when phenotypic data are available through such databases, integrating and reusing those data is a labor-intensive undertaking [36,37].

A “phenome” is the set of all phenotypes expressed by an organism at all life stages (e.g., physical phenotypes, behavioral phenotypes, etc.). It is analogous to the genome or the proteome, which are the sets of all genes and proteins of an organism, respectively. Thus, phenomics is the study of the phenome and how it is determined, especially in relation to genes and environmental influences. Integrated computational analysis of genotype and phenotype is at the heart of precision medicine [38], evolutionary biology [39], and plant breeding [40]. Due to lack of computable phenotypic data, demonstrated advances are limited in portability to other disciplines. Knowledge of cellular and molecular biology has been revolutionized by the “omics” and were made possible by the huge quantities of standardized, computable data. Phenomics holds similar promise on a whole-organism and multi-organism scale but is limited by the lack of computable data.

Many different research disciplines, such as biodiversity science, environmental science, agronomy, biomedicine, and phylogenetics, document characteristics of organisms and taxa. These characteristics have been referred to as traits, phenotypes, characters, and qualities, sometimes interchangeably or inconsistently within and between disciplines. Characteristics of organisms have been represented using several different data models, terminologies, and perspectives, and we will use terms according to the definitions in Box 1. The methods for representing these concepts have arisen independently to address discipline-specific needs; therefore, each community has developed its own terminologies, design patterns, classes, and properties for representing characteristics, sometimes in isolation. The interdisciplinary nature of major societal problems such as climate change, feeding a growing population, public health, and biodiversity conservation will be poorly served by data infrastructure that builds barriers around data sets by discipline. Clarity is needed on how communities of practice are representing organism characteristics to avoid and break down unnecessary silos.

**Box 1. Definitions of commonly used terms**

**Character or trait**: Any descriptor of an organism that can have multiple states/phenotypes (e.g., “leaf shape”). In the phylogenetics community, characters are a special subset of traits that are important for inferring the process of evolution.

**Character state or phenotype**: The specific state or manifestation of a character or trait in an organism (e.g., “ovate” or “12 cm”).

**Quality**: Any descriptor of an organism and its multiple states. In an ontology, the states are subclasses of the descriptor (e.g., “shape” is a parent class of “ovate”).

**Value**: Numerical measurement of a phenotype (e.g., “12 cm”)

**Specimen**: A physical object collected for research purposes. In this context, an organism, part of an organism, or collection of organisms. Specimens are often accompanied by metadata such as time and place of collection.

**Taxon concept**: A hypothesis about how to group individual organisms into species or higher-level taxa.

**Ontology**: An ontology is a classification of concepts in a field of knowledge, or a domain, such as organisms or anatomical entities. Concepts are hierarchically arranged and formally defined in a human-readable format (using text definitions) and computer/machine-readable format (encoded with a knowledge representation language like Resource Description Framework Schema (RDFS), Web Ontology Language (OWL), and Open Biomedical Ontologies format (OBO). In addition, the relationships between concepts are defined, which allows disparate data types to be connected in a formal way.

## Results

### Challenges in phenotypic data sharing

**Fig 1:**
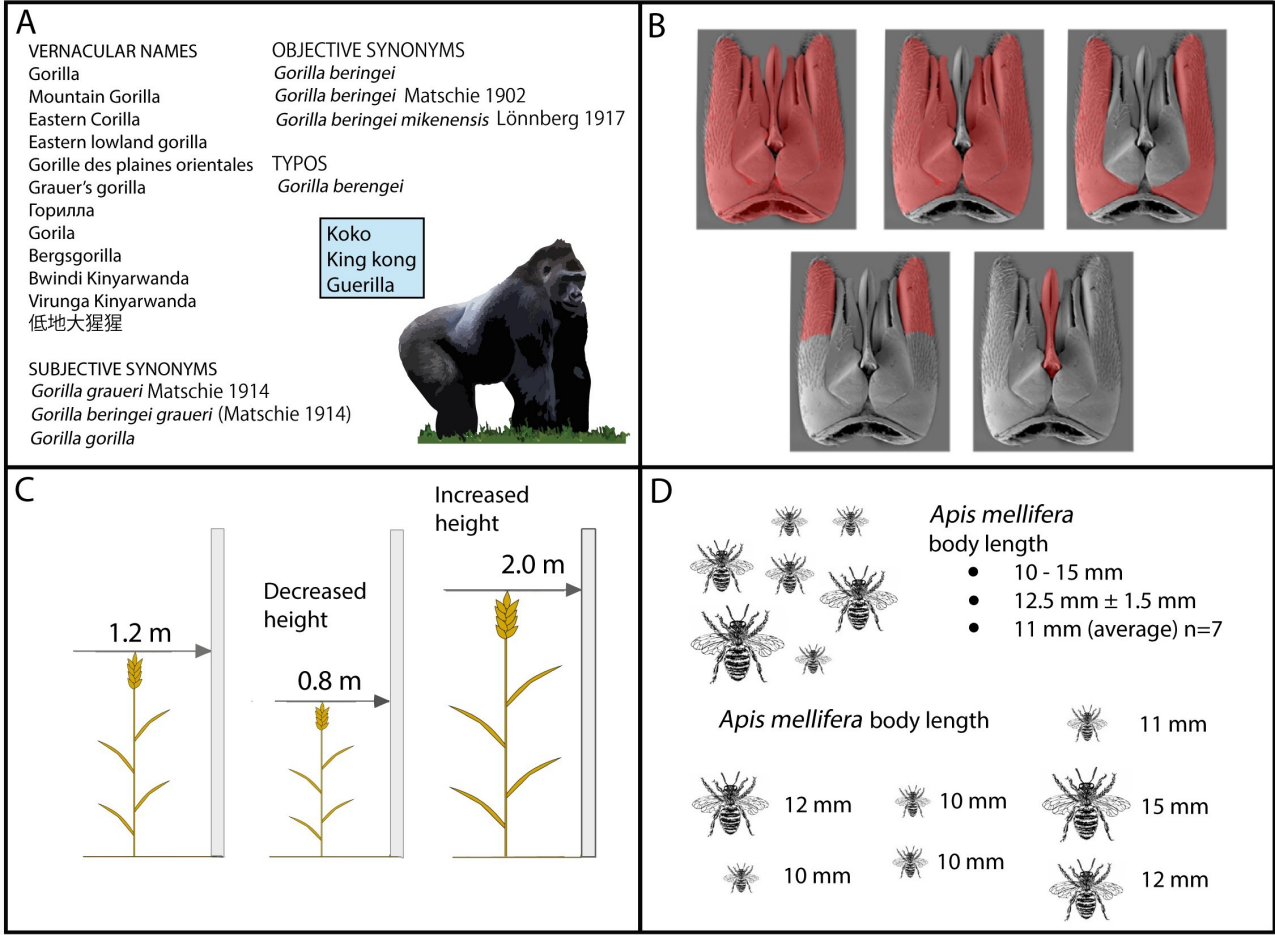
Phenotypic data integration challenges. (A) The many names for the mountain gorilla, *Gorilla beringei*, resulted from years of nomenclatural acts, misspellings, and the quirks of human language and popular culture. (B) The term “paramere” has been ambiguously used to describe 5 different parts of the male genitalia of a gasteruptiid wasp (red). (C) The end-of-season height of a wheat plant can be described by an exact measurement or relative to a “wild type.” (D) With the exception of microorganisms, measurements are collected from specimens but are sometimes represented as a single value representing an entire population or taxon. All 4 of these panels represent 1 or more challenges to phenotypic data integration. *Image credit*: *Panel A by David J*. *Patterson*, *used with permission*.

There are multiple challenges to making phenotypic data available and interoperable, including variable formats, lack of digitization, and linguistic problems such as ambiguity and poor language translation [33,37,41]. We highlight **7 barriers** that stand in the way of integrating phenotypic data:

1. **Many names for 1 thing, 1 name for many things** (Fig 1A and 1B). Several knowledge bases tackle this problem through their own preferred terms and/or controlled vocabularies, sometimes with the addition of a list of synonyms. From an ontological and data integration perspective, this practice presents difficulty, as tracking what preferred term belongs to which organizational context becomes difficult. Similarly, challenges arise when dealing with different languages that use different labels for the same object (e.g., “durum” is “blé dur” in French but “hard wheat” (a type of wheat classification) also translates to “blé dur”). Likewise, people use the same term to refer to many different things [42]. For example, an insect wing, a bat wing, and a wing on an agricultural implement are all very different structures sharing but a few characteristics (e.g., shape), yet they are all called “wing.”
2. **Definitions change over time** (Fig 1B). Nomenclature drift is the process by which the meaning of a word or phrase changes over time. From a knowledge management perspective, as the scale of the information model grows, the thing, the name of the thing, and the definition of the thing must be considered separately in order to deal with real-world complexity, including drift [43]. For example, our concept of a “gene” has changed from a heritable unit, to a coding region of DNA, to being inclusive of all the regulatory regions and potential transcript variants. In ontologies, best practices can control drift by using term identifiers that are independent of the label, ensuring clear textual definitions, requiring a new term identifier when a definition changes meaning substantially, and versioning the ontology; however, it is still very difficult to pinpoint in time when such changes are needed. In less formal vocabularies, there is often no way to control for nomenclature drift. Slang, interdisciplinary pidgin, discipline-specific jargon, and context-specific vernacular ensure that meanings will change over time as organizational cultures shift.
3. **Variable observation granularity** (Fig 1B). One data set may describe the phenotype of an entire leg, while another describes the same phenotype for different parts of the leg. This challenge is only exacerbated when cells, organelles, and behaviors are included. For example, any anatomical part can be partitioned in countless ways, so we need methods and techniques that allow machines to actually evaluate whether descriptions differ because they refer to different objects or just because they (a) focus on different resolutions/scales; (b) use different levels of generality (author 1 refers to a particular cell just as “cell,” whereas author 2 refers to it as a neuroblast); (c) take in different frames of reference; (d) describe different parts of the same object [44,45]; or (e) focus on the same structure at different developmental stages.
4. **Variable perspective**. Describing an anatomical feature from a functional frame of reference will yield a description that is substantially different from a description based on a spatial, developmental, physiological, behavioral, or evolutionary point of view.
5. **Heterogeneous data types** (Fig 1C). Phenotypes are reported using a wide variety of data types, including qualitative, quantitative, relative, or absolute values; and those values can take the form of a boolean, string, integer, or real number. In some data sets, traits are measured quantitatively as absolute integers, such as “rye height = 10 cm” or “petal number = 5,” or boolean, such as “swim bladder = False.” Other data sets report phenotypes qualitatively as a string, relative to some canonical type (e.g., “hind leg enlarged”). This is especially true when dealing with vernacular narratives or field observations of citizen scientists and local experts. It is also the common practice for model organisms, where phenotypes associated with a genetic variant are described relative to the wild type. The integration challenge presents when the same phenotypes are presented as heterogeneous types in different data sets, (e.g., rye height = 10 cm and rye height = stunted and rye height stunted = True).
6. **Specimen versus species or group data** (Fig 1D). All phenotypic measurements for macroscopic organisms are taken from individuals, but they are not always reported or used as such. Often, measurements from 1 or more organisms are pooled and taken to represent the entirety of a group to which the specimens belong, the species, a higher-level taxonomic group, population, or other feature, like sex or life stage. Microscopic organisms can have phenotypes reported per individual or per “strain” if in the laboratory. As a result, data can be reported as coming from individuals (specimens), groups of individuals, or populations (strains or species). This difference in the collection and application of data can result in “average” phenotypes describing taxa that are not qualified with the provenance of a sample size or measure of variation. This is highly problematic in a system that relies on precision, because there is no way to account for error.
7. **Taxonomy changes** (Fig 1A). Circumscriptions of species are hypotheses, as such their definitions also change over time as they are tested, rejected, and refined. The hypotheses that define how species should be defined, i.e., meta-hypotheses, are numerous, and also change over time [46]. The nature of this scenario is not a failure of taxonomy to uniformly address a problem; it is a reflection of the vast complexity observed in biology. The overall fluidity inherent in taxonomy is a strong argument for tying phenotypic data to the specimen or instance level, for if they are reported only at the species level then it is impossible (or at least inadvisable) to interpret those data when species definitions invariably change.

Solutions exist for these barriers, but they require changes to the ways data are collected and managed. One powerful solution is to formally represent phenotypic data using explicit semantics, with a language such as OWL (Web Ontology Language). By logically defining the phenotype concepts, providing text definitions and synonyms, ontologies solve the problems of homonymy, synonymy, polysemy, and most importantly, ambiguity (barrier 1 and 2). For example, the biomedical Natural Language Processing (NLP) community has developed several tools that use reference ontologies (in addition to other resources) for addressing these “word sense disambiguation” problems, enabling a machine to extract meaning from human-readable text [47–49]. The use of ontologies (e.g., that specify that a “tibia” is a “part of” a “leg”) can facilitate integration of data collected at variable scales (barrier 3). Likewise, ontologies that use logical definitions to maintain multiple hierarchies address the challenge of variable perspective (barriers 3 and 4). For example, in the UBERON anatomy ontology [50], a “femoral ridge” is a subclass of both “mesoderm derived structure” (a developmental perspective) and “skeletal element projection” (a spatial perspective). From a functional perspective, any given skeletal ridge might be a subclass of “attachment site.” Ontological models can also be used to combine qualitative and quantitative phenotypic data (barrier 5), but doing so is not straightforward for all phenotypes. For example, the Plant Phenology Ontology (PPO) [51] can integrate count data with categorical data about seasonal changes in plant structures such as leaves and flowers. To give a simplified example, reasoning software could use the PPO to recognize that a quantitative observation of “flower count = 3” also implies the qualitative observation that “flower = present.” Other kinds of quantitative data can be transformed into semantic qualitative annotations by a variety of systematic processes, such as having values with a standard deviation converted into traits indicated as “large” or “short,” while preserving the original values in the knowledge base [52,53]. Providing that the qualitative equivalences are translated in a consistent way, the resulting intercontinental data sets allow for large-scale analyses that were previously impossible. Overcoming the final 2 barriers (barriers 6 and 7) requires that phenotypic data be recorded and preserved at the level of the specimen. Since nearly all phenotypic observations of macroscopic organisms occur at the level of the individual, the challenge is to preserve that level of information in publication. For example, a researcher can collect seven insect body length measurements in a single data set, but might report those lengths in a publication as a mean value (Fig 1D) and that published mean might be the only data that survives long term [54]. Whenever data are aggregated (e.g., reported as a species mean), standard deviation, sample size, and range, if included, would allow these data to be used in future modeling efforts. Preserving specimen-level data also allows phenotypic data to be reassigned whenever species boundaries change. At the microscopic level, it is prohibitively difficult to describe some types of phenotypic data at the individual level. For example, microbial functions, such as the production of certain proteins, are measured on bulk environmental (e.g., soil or water) samples and therefore represent the product of a population or even community. These phenotypes can often be assigned to particular strains but not to a single microbe. In this case, preserving information about the specimen (where it came from, any treatments) becomes crucial for data integration.

Although model organism phenotypic data are rarely described as corresponding to a specimen (and such specimens are rarely preserved), these data are always associated with a genotype. While such data are not foolproof against future changes in taxonomy, the relationship to a known genotype does provide higher precision than simply a species name and facilitates combining model organism phenotypic data with data from nonmodel species (e.g., [55]). The use of ontologies to describe phenotypes is common in the model organism domain. Decades of work in this community have resulted in a massive body of interoperable data that has had a real impact [56–58]. We have every reason to believe that a comparable effort in other disciplines would be just as impactful.

### Approaches to making phenotype definitions computable

There are several databases containing information about organism characteristics (Table 1). All of the repositories and models discussed here will be grounded in some type of pattern, based on the way they use ontologies. For a comprehensive and continuously updated list of trait data repositories, see the Open Traits Network [37,59]. Standards and models are just as much a product of the state of the user community as they are an expression of an efficient way to represent data. This is apparent in many of the differences between the models discussed below.

**Table 1:** Semantic knowledge bases containing information about organism characteristics.

Name*	Description or Scope	Format	Pattern	Reference
Biodiversity				
Phenoscape^†^	Vertebrate morphology	OWL in RDF Blazegraph triplestore	EQ	[35,60]
EOL TraitBank^†^^‡^	Internet aggregator of data about species	Neo4j	Character/Character State	[61,62]
Microbial Phenotypes Wiki^‡^	Web-based community resource designed to display microbial phenotypes and the methods used to study them.	MediaWiki	Tabular, uses OMP [63]	[64]
PolyTraits^‡^	Database on biological traits of polychaetes	Relational database	Character/Character State	[65,66]
TRY^†^	Global database of curated plant traits	Relational database	Map traits to TOP (EQ) [67]	[34]
FuTRES^†^	Functional traits of vertebrates	OWL in RDF triplestore	Measurement-Based quantitative data, trait definitions follow EQ pattern from OBAEQ	[68]
Planteome^‡^	Plant genomics and phenomics	GAF and SOLR	EQ and DOS-DP	[69]
Global Plant Phenology^†^	Aggregator of plant phenological data	OWL and JSON	Measurement-Based quantitative and presence/absence data; EQ model	[70,71]
Semantic Morph·D·Base^†^	Repository for morphological data	OWL in RDF triplestore	Measurement-Based with connection to TBox: Phenotype Knowledge Graphs	[72–76]
TaxonWorks^†^	Web-based workbench for taxonomists and biodiversity scientists	PostgreSQL (relational database)	Class (OTU) or Measurement-Based (collection object). Qualitative, quantitative, statistical, media, gene, text, presence/absence, arbitrary triples (data attributes).	[77]
World Register of Marine Species^‡^	Authoritative classification and catalogue of marine species	MS SQL relational database with trait module	Character/Character State	[78]
Agriculture				
Gramene^‡^	Comparative functional genomics in crops and model plant species	MongoDB	JSON-like, using PO [79]	[80,81]
Sol Genomics Network^‡^	Clade-oriented database dedicated to the biology of the Solanaceae family	Relational database (chado)	Tabular, dbxref to PO	[82,83]
GrainGenes^‡^	Comprehensive resource for molecular and phenotypic information for wheat, barley, rye, and other related species, including oat.	Relational database (chado)	Tabular, using Plant TO [84]	[85,86]
Annex^‡^	Cereals ontology	OWL	Measurement and Class-based	[87]
CassavaBase^‡^	Genomic and phenomic resource for cassava	Relational database (chado)	Tabular, uses CO [88]	[89,90]
AgroLD^‡^	Integrated data about commercially important plants	RDF triples	EQ and DOS-DP	[91]
Biomedicine and Model Organisms				
Monarch Initiative, uPheno, and Human Phenotype Ontology^‡^	Integrator of cross species genotype-phenotype data including human phenotypes and their relationship to diseases	OWL	EQ and DOS-DP	[28,92,93]
MGI^‡^	Mouse genomic and phenomic resource	OWL and OBO	EQ and DOS-DP	[94,95]
WormBase^‡^	Nematode genomic and phenomic resource	OWL and OBO	EQ and DOS-DP	[96,97]
TAIR^‡^	*Arabidopsis* genomic and phenomic resource	OWL and OBO	EQ and DOS-DP	[98,99]
FlyBase^‡^	Fruit fly genomic and phenomic resource	OWL and OBO	EQ and DOS-DP	[100,101]
XenBase^‡^	*Xenopus* genomic and phenomic resource	OWL and OBO	EQ and DOS-DP	[102,103]
ZFIN^‡^	Zebrafish genomic and phenomic resource	OWL and OBO	EQ and DOS-DP	[104,105]
Saccharomyces Genome Database^‡^	Comprehensive integrated biological information for the budding yeast *Saccharomyces cerevisiae*	PostgreSQL	Tabular, uses APO [106]	[107]
RGD ^‡^	Structured and standardized data for 8 species (rat, mouse, human, chinchilla, bonobo, 13-lined ground squirrel, dog, and pig)	Relational database (chado), GAF, and OBO	Qualitative, links QTLs to multiple OBO phenotype ontologies	[108,109]

*To be included in this table, a resource must contain annotations linking traits to organisms, use a phenotype ontology, and not require login credentials.

^†^Includes phenotype data reported at the individual specimen level.

^‡^Includes phenotype data reported at the group level.

APO, Ascomycete Phenotype Ontology; CO, Crop Ontology; DOS-DP, Dead Simple Ontology Design Pattern; EQ, Entity–Quality; GAF, GO Annotation File format; OBAEQ, Ontology of Biological Attributes-Entity Quality; OBO, Open Biomedical Ontologies format; OMP, Ontology of Microbial Phenotypes; OWL, Web Ontology Language; OTU, Operational Taxonomic Units; PO, Plant Ontology; QTL, Quantitative Trait Locus; RDF, Resource Description Framework; RGD, Rat Genome Database; TO, Trait Ontology; TOP, Thesaurus of Plant Characteristics.

### Classes versus instances

**Fig 2:**
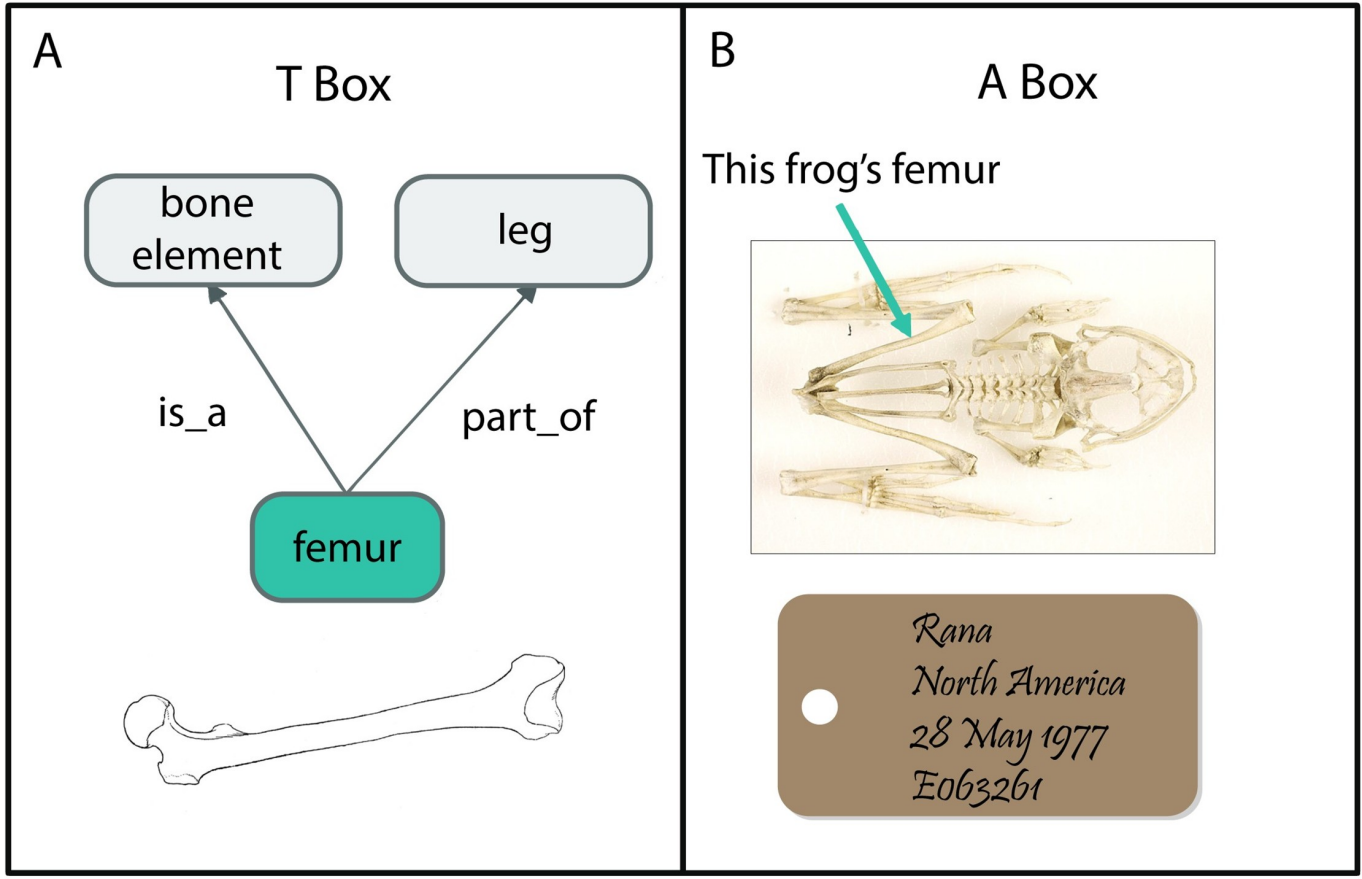
TBox versus ABox. The TBox (A) includes classes (kinds of things), properties (the possible relationships between classes and instances of the classes), and assertions about the classes and properties. The ABox (B) represents instances of the classes represented in the TBox and assertions about those instances. For example, an instance of femur in a frog specimen is 1.2 cm long. *Image credit*: *Photo from National Museum of Natural History*, *Washington DC*.

An important concept in understanding the diversity of phenotypic data is the recognition that some assertions are made at the “class” level (e.g., types of things) and others at the “instance” level (e.g., individual organisms or their parts). TBox reasoning is defined as logical entailments regarding axioms about classes and properties, whereas ABox reasoning utilizes axioms about instances (Fig 2). The terms “TBox” and “ABox” are used in computer science and refer to the terminological component and the assertion component, respectively. “TBox” refers to classes, properties, and assertions about those classes and properties that are true in the general sense; for example, that a human femur is a type of bone and is a part of a leg—this is true for all instances of femur, bone, and leg. “ABox” refers to instances of classes and assertions that are instance specific, for example, that a specific organism’s femur is 12.4 cm long. These 2 levels of knowledge express different kinds of truths and require different representational models.

In biological domains, the TBox is often implemented as an ontology with “classes” that describe kinds of things, like “femur,” “leg,” and “bone,” and “properties” that describe the relationships among the classes or relationships among instances of those classes. The properties have machine-readable rules to describe how the kinds of things relate to each other (and globally unique, persistent identifiers to that a vertebrate femur and an insect femur are not conflated). So, in the example above, that “femur” “is a” type of “bone” and “part of” a “leg;” “femur,” “leg,” and “bone” are classes, while “part of” and “is a” are the properties. The ABox uses the classes and properties described in the ontology to model instance data. The bridge between the TBox and ABox is the use of common classes, in this case, “femur.” In the ABox, the data represent instances of the class “femur,” which is described in the TBox.

### Semantic phenotypes encoded using Entity–Quality Formalism

**Fig 3:**
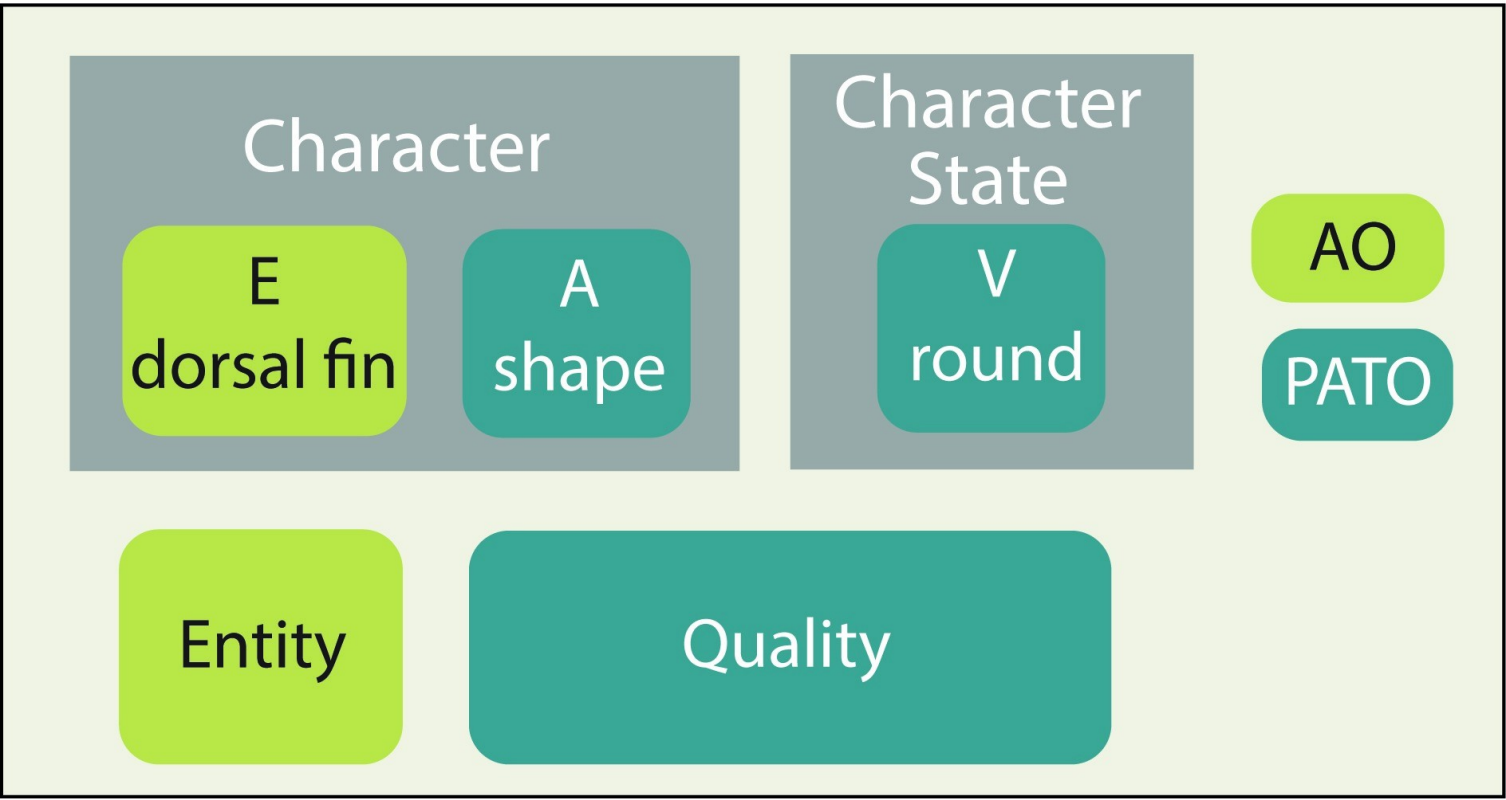
EQ Formalism for categorical phenotypes versus character states. From [112]. The EQ Formalism uses ontology terms from an anatomy ontology (green) and a trait ontology (blue) to represent a phenotype and maps to the Character/Character State model (gray). EQ, Entity–Quality.

Entities in ontologies may be defined by or composed of multiple other classes. For phenotypes or traits, this is done using the Entity–Quality (EQ) Formalism [25]. This model combines terms from anatomy ontologies (entities) and phenotype ontologies (qualities) to make an abbreviated assertion that an anatomical entity has a particular quality (Fig 3). Entities need not be anatomical and can include processual entities (e.g., E = “migration,” Q = “delayed”) or physiology (e.g., E = “transpiration rate,” Q = “increased”). With additional modeling, values, such as a numerical measurement of body length, can also be included [52,53]. This approach to representing phenotypes originated largely in the model organism community and has been adapted to translate phylogenetic matrices into machine-readable assertions [25,110,111]. While the EQ examples given here are straightforward, phylogenetic characters can sometimes require very complex EQ statements because they were historically not developed with formal logic in mind and may include multipart character states.

When such EQ-based traits are made into named classes, they are said to be “pre-composed” (e.g., the “flower color” trait from the Plant Trait Ontology (TO:0000537), which is axiomatized as [“color” and (“inheres in” some “flower”)] where “color” comes from Phenotype and Trait Ontology (PATO), “inheres in” comes from RO, and “flower” comes from PO). Alternatively, one could assert that an instance of the “length” class is a quality of an instance of the “femur” class without making a new, named class. In this latter case, femur length is “post-composed.” These 2 approaches are logically equivalent and can be reasoned over (with strategically placed equivalence axioms) [25]. The choice to pre- or post-compose entities will depend on the use case and resources available for maintenance. When a defined concept is likely to be used many times, or if it is itself part of more complex entity definitions, then pre-composition can assure that it is defined and used consistently each time. For a project that requires defining many types of traits combinatorially (e.g., any material type with any quality), with zero or few instances of each combination, post-composition may work better but is a more manual process and requires a good user interface and logical consistency rules. The EQ Formalism can be used to construct pre- or post-composed classes, depending on the use case.

Categorical phenotypes, for example, “shortened femur” or “delayed germination,” are often described as relative to some wild type (e.g., associating “shortened femur” with a genotype implies that other genotypes have longer femurs) and can readily be represented using EQ Formalism. As described above, the EQ Formalism can precompose phenotype classes using an anatomy or process ontology and the PATO [113]. Qualitative phenotypes expressed as EQ have been connected to genes, variants, and other annotations using the GAF file format [114]. The combination of logical axioms and annotations that relate phenotypes with other biological entities in a computable graph can be analyzed by reasoning software and semantic similarity algorithms to answer questions. This method has been used for inferring candidates for disease diagnosis [56] and identifying genes responsible for anatomical evolution [55]. Analysis of categorical phenotypes is very different from the analysis of quantitative phenotypes but just as valuable.

More recently, several phenotype ontologies implemented the Dead Simple Ontology Design Pattern (DOS-DP) ontology building process to consistently precompose classes and represent more granular phenotypes [25,115]. This combination of EQ and DOS-DP creates consistent and reusable phenotype ontology classes. For example, the “femur” class in UBERON and the “length” class in PATO can be combined in a more specific trait ontology such as Ontology of Biological Attributes (OBA) to make the precomposed class “femur length” [length and (inheres in some femur)]. Plant ontologies follow a similar pattern using “flower” from PO and “shape” from PATO to construct “flower shape” in the Plant Trait Ontology (TO) [84] [shape and (inheres in some flower)]. Additional logic is needed to include a qualitative assessment of the phenotype. For example, a “shortened femur” phenotype class would use “decreased length” from PATO and “femur” from UBERON [(decreased length and (inheres in some femur) and (has modifier some abnormal))]. EQ Formalisms and DOS-DP create consistent, logically defined phenotype classes that can be made available with minimal maintenance cost. Without these simple design processes, ontology developers can find themselves overwhelmed with revisions and alignments as updates reverberate through a complicated, interconnected ontology. As a result, this pattern has seen relatively wide adoption and has been used for basic research and applied purposes. For example, Phenoscape [60] uses post-composed EQ Formalisms for identifying the underlying genetic basis of evolutionary change [55]. Through the use of EQ Formalisms, the Planteome project [69] allows users to identify the genetic basis of crop diversity and differential response to environmental conditions [116]. The Monarch Initiative [28], which includes uPheno [117] and the Human Phenotype Ontology project [93], uses precomposed EQ Formalisms for revealing genetic basis of disease and aiding diagnosis [118]. Several model organism databases [94–105] use EQ Formalisms to document genotype–phenotype associations in an interoperable way, and the TRY Plant Trait Database [34] uses them to support global integration and analysis of functional biodiversity in plants. These resources all use ontologies developed within the OBO Foundry [119] with a set of basic development principles that helps ensure logical consistency across projects. This kind of interoperability is what enables more complex patterns that support computable representations of phenotype.

### Character/character states

**Fig 4:**
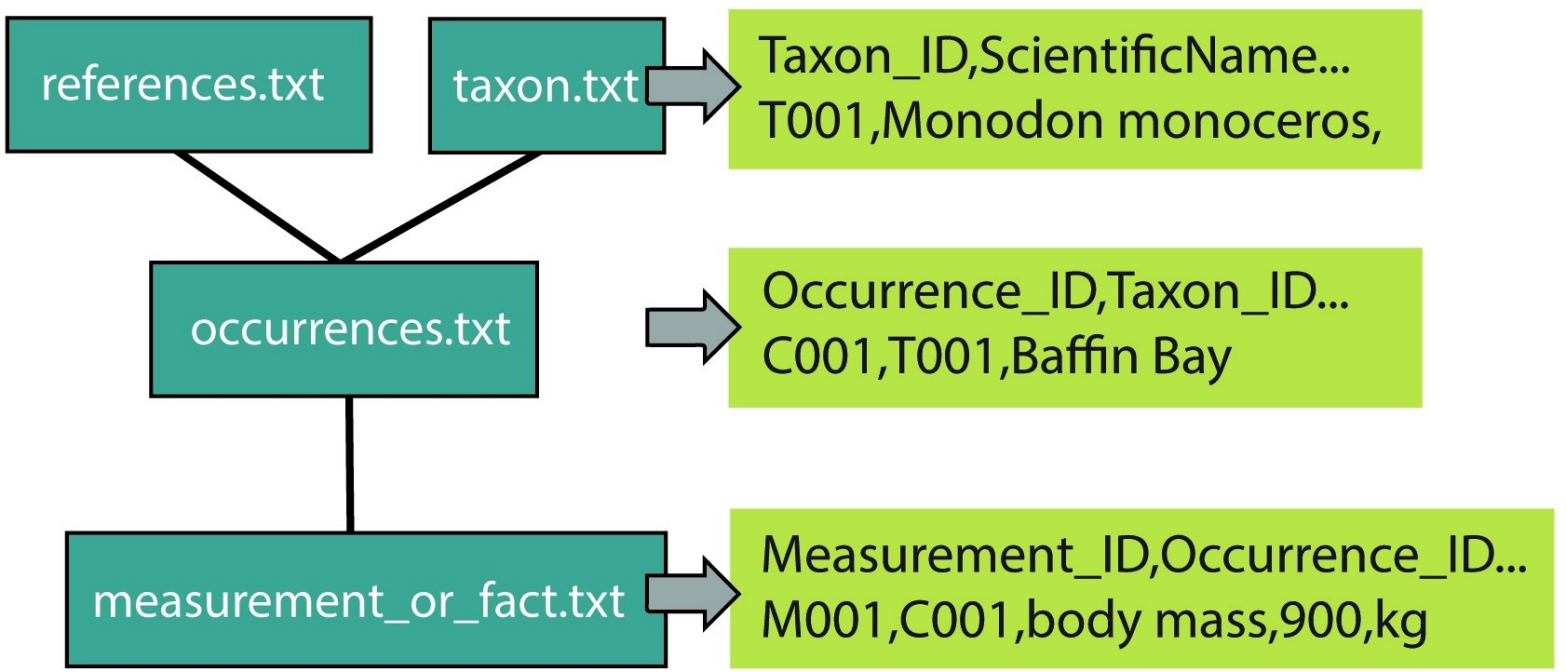
Darwin Core star schema with traits. Phenotypes can be represented in the Darwin Core star schema that consists of separate tabular files (blue) linked together by unique identifiers for taxa, occurrences, and measurements (green).

The Character/Character State semantic model reflects the long history of research strategies and data structures in systematics, taxonomy, and phylogenetics, which is dominated by tabular data exchange standards. Phylogenetic data (e.g., dorsal fin shape—round) are represented in a matrix for tree-building algorithms (e.g., Mesquite [120]). Taxonomic and systematic data are represented in a table according to a biodiversity data exchange standard, such as Darwin Core, which was recently adapted to include a measurements and facts extension [121,122] and represented as a property graph [123] (Fig 4). If we translate this tabular standard directly into a semantic assertion, we necessarily include 3 classes, for example, “dorsal fin,” “shape,” and “round” (Fig 3). Both characters and character states come from an ontology like the OBA (characters) or PATO (character states), but this model is not fully computable because the relationships between the classes in the data set are not defined by an ontology. This model can include characters other than strict phenotype information, such as habitat types or trophic strategies.

This model is quite flexible and capable of accommodating historical data. The biggest advantage to this model is its compatibility with the existing infrastructure of biodiversity databases that are using the Darwin Core standard. The “spreadsheet” data format is familiar to many researchers, citizen scientists, and local experts, unlike OWL. The use of ontology classes to define the characters and character states aids in translating these data from tables to graphs; however, there are limitations in translating from class-based to instance-based because of the difficulty in retaining specific collection metadata in the class-based approach [75]. The Character/Character State model is used by EOL TraitBank [124], the World Register of Marine Species (WoRMS) [78], and PolyTraits [66] to provide phenotypic data in conformance with existing biodiversity data exchange standards. These 3 resources are considered content aggregators who bring together information from distributed data sources to present to a user in a unified platform, such as a web site. Many of these larger aggregators, like WoRMS and EOL, need similar data types for millions of taxa across the tree of life, which means that they prioritize broadly applicable traits, like mass, and sometimes must use data with less detailed metadata, like central tendency of a taxon mass rather than population mean with standard deviation. In addition to data aggregation, EOL uses a simplified ontological structure as a content navigation and access tool on their web site. The flexibility of this model and its conformance to existing biodiversity standards meets many of the content acquisition and delivery needs of aggregators like EOL, WoRMS, and PolyTraits.

### Measurement-based quantitative phenotypes

**Fig 5:**
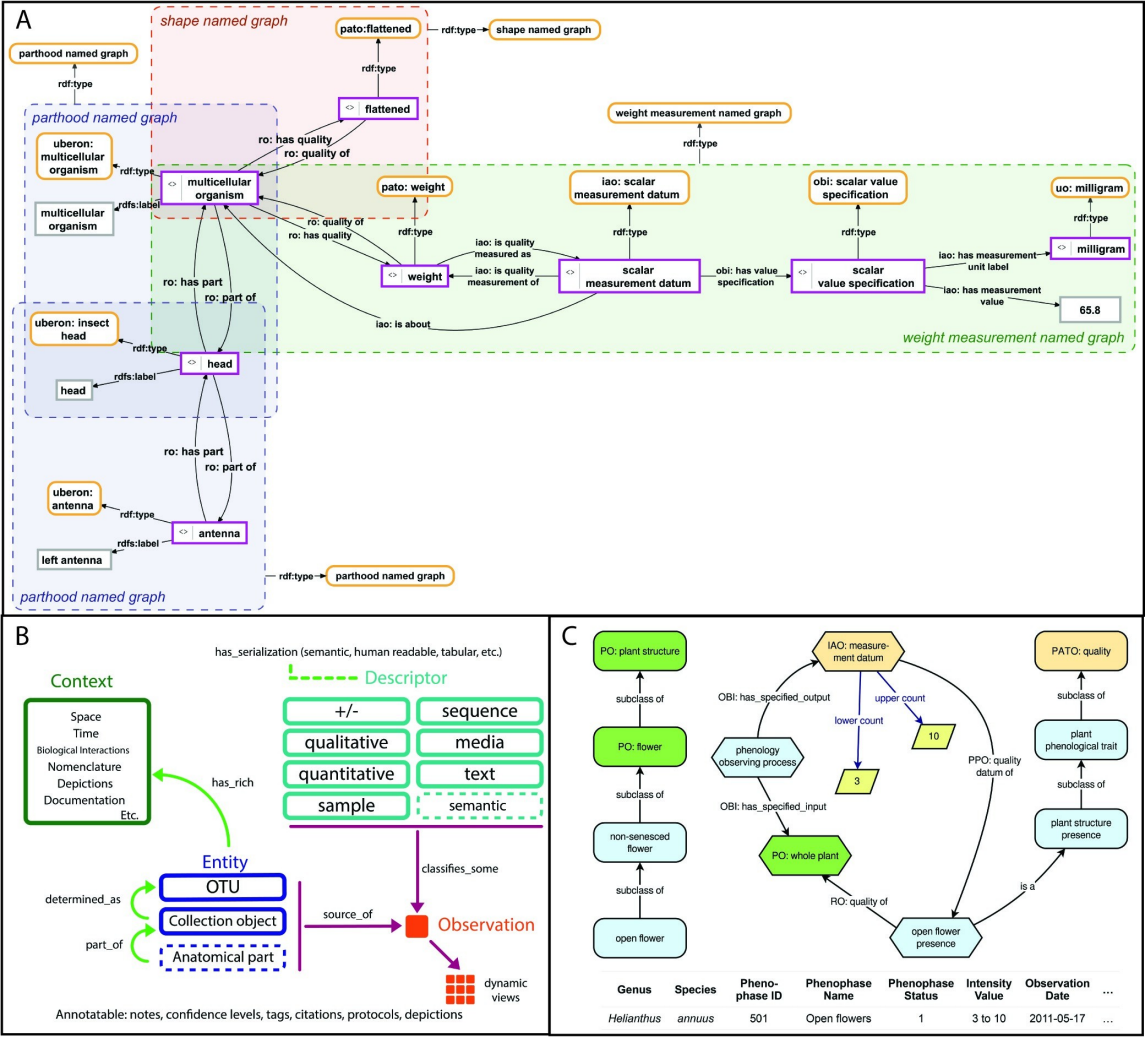
Measurement-Based phenotype data models. (A) Semantic Morph·D·Base. Pink-bordered boxes: instances; yellow-bordered boxes: classes; gray-bordered boxes: literals (labels or values); boxes with dashed borders: named graphs. (B) TaxonWorks. The underlying goal is to let scientists assert phenotype observations as required for their research. Assertions are persisted in Descriptor–Observation format where subclasses of descriptor (e.g., qualitative, quantitative, statistical, gene, free-text, and media) classify/define observations. Descriptor types anticipate downstream serialization into computable formats, semantic or otherwise. Phenotype assertions are at the class (= Taxon concept, an “OTU” in TaxonWorks) or instance (= Collection object) level (“Entity”). Ultimately, both levels will permit anatomical part assertions. While the approach includes improvements to the overall semantics, it still lacks specifics used in other models (e.g., Fig 5A and 5C); however, the typed descriptor approach provides a flexible software design, whereby incremental improvements to semantics are possible. All data are highly annotatable. Dashed boxes are features in progress. (C) Global Plant Phenological Database. Rounded rectangles represent classes, and hexagons represent instances. The original data set (bottom of figure) indicates that there is an instance of the class/phenophase “open flower presence,” which is a quality of an instance of “whole plant” from the PO. Because the value of the instance of measurement datum is >0, the ontology infers that open flowers are present. Due to the subsumption hierarchy of the PO (left side of figure), the ontology can also infer that nonsenesced flowers, flowers, and plant structures are present. IAO, Information Artifact Ontology; PATO, Phenotype and Trait Ontology; PO, Plant Ontology; OBI, Ontology for Biomedical Investigations; OTU, Operational Taxonomic Unit; RDF, Resource Description Framework; RO, Relations Ontology; UO, Unit Ontology.

This model is an extension of the EQ Formalism that accommodates measurements from individual specimens and is frequently utilized in tools for users to record observational data (Fig 5). Details about who, how, when, and where measurements were made can be modeled using the Information Artifact Ontology (IAO) [125] and the Biological Collections Ontology (BCO) [126]. The BCO was originally developed as a model for occurrence data stored in Darwin Core Archives but was later expanded to encompass observations, which produce trait data [126]. Unlike Character/Character State methods, which use a tabular data structure, Measurement-Based methods use a graph data structure, although tools exist for converting data from tables to graphs [127]. While the EQ Formalism can contribute to a Measurement-Based model, the latter often includes extensive metadata about the measurement process that EQ, at its most basic, does not include. As a consequence, the graph containing the description can be fragmented into subgraphs based on user need. Existing phenotype descriptions can also be easily expanded with additional information by simply adding further triple statements. Another consequence is the possibility to assign differentiated metadata to various subgraphs of a description, which allows, for example, tracking different sources of evidence used in a description or information about who contributed to which parts of the description [48]. Measurement-Based quantitative phenotypes are used by Semantic Morph·D·Base [72–75] to describe specimen phenotypes with very specific collection metadata, TaxonWorks [77] to allow researchers to assert phenotype observations as needed in taxonomic research, the PPO and Global Plant Phenological Database [70,71] to integrate citizen scientist observations for large-scale analysis of phenology, and FuTRES [68] to describe specimen phenotypes for meta-analysis. All of these platforms are designed for an expert user to manage detailed observation data for research applications.

The largest volume of Measurement-Based assertions about organism phenotypes comes from the clinical domain. A single person may have thousands of assertions about them and be in a collection of hundreds of thousands of people. A comprehensive list of these repositories and their data models is outside the scope of this manuscript.

## Discussion

Representing organismal characters in a machine-readable form brings modern data science and computational power to the study of organismal diversity. The most common structured representations of phenotypic data are a phylogenetic matrix, data table, or semistructured text, but most phenotypic data are semistructured in hard-to-find supplementary files or unstructured in narrative text or images [128]. Much of the structured data currently in knowledge bases were hand curated (e.g., [129]). This means that the majority of the work to integrate phenotypes at scale is in digitization and mining rather than developing semantic models. Various strategies for automated mining of phenotypes from text, images, specimens, and character matrices have been developed, but most are very specific to a type of text or taxon and require significant curation of results [130–133]. Tools have been developed for the semiautomated translation of phylogenetic matrices and text descriptions into semantic statements that have been successfully used to populate knowledge bases [134,135]. While much progress has been made in the development of tools for translating trait data into a semantic structure, most phenotypic data are only available in human-readable form.

The community has not yet reached a consensus around how phenotypic data should be modeled in semantic knowledge bases using ontologies; however, some discipline-specific best practices have been developed. The Biolink model has been an effective meta-model for integrating phenotypic data across biomedical knowledge graphs that could potentially be used in other disciplines [136]. The Minimal Information About Plant Phenotyping Experiment (MIAPPE) provides best practices for recording agricultural phenotyping data that can be adapted to other types of organisms [137]. The Investigation/Study/Assay tab-delimited (ISA-TAB) format is a framework to represent complex metadata from “omics-based” experiments that can be represented semantically [138]. The Generic Model Organism Database project (GMOD) developed the chado database schema, which provides shared tools, services, and ontologies [139–141]; however, many of the GMOD repositories use a specialized trait vocabulary or ontology that is not linked to any other phenotype ontology (e.g., MaizeGDB [142], Bovine Genome Database [143], SoyBase [144], and VectorBase [145]). Despite these efforts, ontologically supported knowledge bases are still less popular than other data structures such as relational databases and tabular data files. The lack of tools and services for the management and curation of phenotypic data combined with the high degree of technical expertise required to cope with the complexity of semantic modeling is likely a major reason for this lack of adoption. Despite this, there is general consensus building around the need for a shared phenotype model, the use of terms from ontologies, and standardized methods for capturing trait observations [37].

It is unlikely that existing knowledge bases will be able to quickly redesign their systems to adopt a new, unified model; thus, it becomes important to map across the different models. Translation from 1 model to another can be straightforward, especially if shared ontologies or a meta-model, such as Biolink, are used. Difficulty arises when trying to combine or transform phenotypes reported at the individual organism level with phenotypes reported at the group level. When possible, phenotypes should be reported at the individual level because these can be aggregated to calculate a group-level phenotype. Breaking up group-level phenotypes into the more granular individual-level phenotypes is not possible. One possible exception is the reporting of traits for strains, cultures, or cultivars, wherein all individuals are supposed to be genetically identical (but this is not always the case). In addition, reporting phenotypes for individuals then allows integration of the phenotypes with any other metadata collected about that individual, such as its environment, biotic interactions, or genotype. While recording data at the individual level is preferable to the group level, this is not always possible for existing knowledge bases with established data models and a method for mapping across models is needed.

Ontologies and semantic knowledge bases provide a way to overcome barriers to integration at scale but are limited by the lack of supporting infrastructure to make them easy to use in practice, which requires a balance between human usability and computational capabilities. Essential usability components include provenance tracking and documentation of design decisions and the collaborative decision-making process [146,147]. Resolution of the complex conflicts that can occur as the size and scope of a knowledge base or ontology increases, depends on third parties being able to understand design decisions, sometimes years later. Useful provenance tracking and documentation can be achieved using Minimum Information for Reporting an Ontology (MIRO) guidelines [147], established best practices for defining and labeling ontology classes [148,149], provenance and attribution ontologies such as the Provenance Ontology (PROV-O) [150], Scientific Evidence and Provenance Information Ontology (SEPIO) [151], and Contributor Role Ontology (CRO) [152], and the built-in versioning and issue tracking in environments like GitHub. Terminology registries such as BioPortal [153], Linked Open Vocabularies [154], and AgroPortal [155] aggregate important provenance information and metrics that can aid the user in finding an appropriate ontology. A portal like Ontobee [156], which shows how classes are used in logical axioms across several ontologies, helps the user understand how to use a class in creating an EQ Formalism, for example. The availability of ontology registries enables knowledge engineers to record ancillary data in a machine-accessible manner. This is important because as the complexity of a knowledge base grows so does the amount and variety of ancillary data, that would otherwise be entered as a string in a comment field. Lastly, repositories also provide a forum for the community evaluation of the ontologies while supporting the discovery of other actors and projects that have the very same specific ontological domains.

Lastly, the complexity of ontologies requires documentation in addition to standard approaches [157–159], such as term definitions targeted to different audiences, e.g., domain experts, ontology engineers, and developers. The complexity of semantic structures makes documentation that reflects both the contents and organization of the system, in addition to the intent of the designers, a requirement for long-term, sustainable use of ontological resources. Essential to the success of an ontological resource or knowledge base is a vibrant user community, which requires infrastructure to support active engagement of the communities these semantic resources are meant to serve. The value of proper documentary procedures and provenance information cannot be overstated in the ontological field as they provide the ability to justify axioms, permissible intellectual property usage, and the authoritativeness of the information used to build the ontology.

This paper discusses 3 different phenotypic data models, EQ Formalism, Character/Character State, and Measurement-Based. The EQ Formalism and Measurement-Based models are closely related in that they both have significant logical semantics. The Measurement-Based approach links specific values to specimens, rather than linking averages to taxon concepts, and thus is more easily adaptable to taxonomic changes that can rearrange the assortment of specimens (and their phenotypes) within taxa. Conversely, the Character/Character State model is much more straightforward for individual researchers, is more closely conformant to existing biodiversity standards, and can represent qualitative and quantitative data for a class or an instance in a similarly straightforward manner. As a result, numerous data sets are developed for aggregators using the Character/Character State model that may not be made conformant to any other standard. Thus, we recommend development of a workflow for passing data from individual researchers to content aggregators using the Character/Character State model that can be translated to OWL semantics using a Measurement-Based or EQ approach. Such a workflow would include transforming small data sets to conform to an aggregator standard (similar to the process EOL TraitBank currently uses) and then transformation of these data into OWL semantics. Significant work is required to develop the infrastructure to support this workflow including expanding the coverage of ontologies and semantic data models, developing an interface for data access, and creating a governance model for long-term sustainability and maintenance of the resource. This workflow is dependent on source data sets being properly licensed for sharing and reuse [160], which may require significant negotiation [161]. Such an effort would be hugely valuable for phenotypic data integration and the capture of “dark data” [31]. In this context, we agree with the open science principles put forward by the Open Traits Network [37], especially the development of a “trait core” that can apply life-wide, and would add the recommendations to build on the existing meta-modeling efforts of the Biolink model and the ontology design patterns successfully being used within the OBO Foundry family of ontology projects. These standards have already been successfully used to overcome the barriers to data integration listed above to integrate phenotypic data from model organism databases [28]. Standardization of phenotype representations using DOS-DP [115] directly or indirectly via Biolink model mapping, will take the state-of-the-art from just aggregating distributed trait data sets, to truly integrating them.
